# The Effect of CaV1.2 Inhibitor Nifedipine on Chondrogenic Differentiation of Human Bone Marrow or Menstrual Blood-Derived Mesenchymal Stem Cells and Chondrocytes

**DOI:** 10.3390/ijms24076730

**Published:** 2023-04-04

**Authors:** Ilona Uzieliene, Daiva Bironaite, Rokas Miksiunas, Edvardas Bagdonas, Raminta Vaiciuleviciute, Ali Mobasheri, Eiva Bernotiene

**Affiliations:** 1 Department of Regenerative Medicine, State Research Institute Centre for Innovative Medicine, 08406 Vilnius, Lithuania; 2 Research Unit of Health Sciences and Technology, Faculty of Medicine, University of Oulu, 90014 Oulu, Finland; 3 World Health Organization Collaborating Center for Public Health Aspects of Musculoskeletal Health and Aging, Université de Liège, B-4000 Liège, Belgium; 4 Department of Joint Surgery, First Affiliated Hospital of Sun Yat-sen University, Guangzhou 510080, China

**Keywords:** intracellular calcium ions, voltage-operated calcium channels (VOCC), mesenchymal stem/stromal cells (MSC), chondrocytes, chondrogenic differentiation

## Abstract

Cartilage is an avascular tissue and sensitive to mechanical trauma and/or age-related degenerative processes leading to the development of osteoarthritis (OA). Therefore, it is important to investigate the mesenchymal cell-based chondrogenic regenerating mechanisms and possible their regulation. The aim of this study was to investigate the role of intracellular calcium (iCa^2+^) and its regulation through voltage-operated calcium channels (VOCC) on chondrogenic differentiation of mesenchymal stem/stromal cells derived from human bone marrow (BMMSCs) and menstrual blood (MenSCs) in comparison to OA chondrocytes. The level of iCa^2+^ was highest in chondrocytes, whereas iCa^2+^ store capacity was biggest in MenSCs and they proliferated better as compared to other cells. The level of CaV1.2 channels was also highest in OA chondrocytes than in other cells. CaV1.2 antagonist nifedipine slightly suppressed iCa^2+^, Cav1.2 and the proliferation of all cells and affected iCa^2+^ stores, particularly in BMMSCs. The expression of the CaV1.2 gene during 21 days of chondrogenic differentiation was highest in MenSCs, showing the weakest chondrogenic differentiation, which was stimulated by the nifedipine. The best chondrogenic differentiation potential showed BMMSCs (*SOX9* and *COL2A1* expression); however, purposeful iCa^2+^ and VOCC regulation by blockers can stimulate a chondrogenic response at least in MenSCs.

## 1. Introduction

Human articular cartilage is a highly specialized, avascular tissue, which has a low ability to repair lesions, which lead to diseases such as osteoarthritis (OA) [1,2,3]. OA is known to be the most common form of arthritis and a leading cause of human disability worldwide, largely due to pain, as a primary symptom of the joint impairment [1,4]. Up to date, the most common treatment includes OA-related pain suppressing non-steroidal anti-inflammatory drugs (NSAIDs) and other types of painkillers [5,6,7]. However, very often the therapeutic result of the conventional treatments is disappointing. Therefore, OA cartilage repair by applying stem cells has been considered as a promising field of articular regenerative medicine [8,9,10,11].

The pluripotent stem cells and the mesenchymal stem/stromal cells have been so far mostly investigated in OA tissue regeneration studies both in vitro and in vivo [12,13,14]. As the human embryonic stem cells (ESCs) have limited applicability due to ethical issues, mouse and other experimental animal ESCs can generate an unlimited source of chondrocytes and other tissues specific cells under the appropriate conditions in vitro [15]. Another type of pluripotent stem cells, induced pluripotent stem cells (iPSCs), have also been intensively investigated in the cartilage tissue regeneration field; however, the challenges of directing their differentiation as well as risks related to the elusive origin and tumorigenesis should not be ignored [16]. Due to the ability to differentiate into chondrocytes and immunomodulatory properties, adult tissue-derived MSCs have been suggested to be one of the most attractive tools for the OA regenerative studies both in vitro and in-vivo [17,18,19]. Rapid proliferation, minor immunological rejection expands the therapeutic application of MSCs in the regulation of OA pathogenesis. Among various types of human tissues-derived MSCs, the adipose [20], human synovial membrane [21], autologous and allogeneic bone marrow [22,23] and chondrocytes [24,25], umbilical cord [26] in patients and animal models have been investigated for OA regeneration purposes. Recently, menstrual blood-derived MSCs have also been shown to have chondrogenic differentiation properties [27]. Therefore, in this study the chondrogenic potential of human BMMSCs, menstrual blood-derived MSCs (MenSCs) and OA cartilage-derived chondrocytes have been studied in vitro, considering the possible further application for the therapeutic OA regenerative purposes in vivo. 

The level of intracellular calcium ions (iCa^2+^) controls a number of signaling pathways important for the cell functioning, proliferation and differentiation including chondrogenesis. An increasing body of evidence shows that calcium ions (Ca^2+^) participate in the regulation of differentiation potential of chondrogenic progenitor cells [28,29,30]. However, the effect of different iCa^2+^ levels, as well as mechanisms of iCa^2+^ regulation in the chondrogenic differentiation of mesenchymal and other types of stem cells are still unclear [31]. The importance of intracellular ion homeostasis in the functioning of chondrocyte [32], as well as other types of cells, has been extensively demonstrated [33]. Plasma membrane-situated proteins such as L-type voltage-operated calcium channels are crucial for the regulation of the iCa^2+^ level and further cell functioning [34]. VOCC are predominantly found in excitable cells, such as neurons, cardiomyocytes, glial or other types of the cells that can be extracellularly stimulated [13,35]. However, VOCC channels can also be expressed in a wide range of non-excitable cells, including chondrocytes [36]. The mechanism of VOCC action goes through the cell membrane depolarization and Ca^2+^ flow into the cells mainly through the VOCC subunits CaV1.2, which regulates the iCa^2+^ level and thus coordinates cell differentiation leading to the proper tissue development and functioning [37].

Chondrogenesis is regulated by the interplay between numerous intra- and extracellular factors [29]. Various cardiovascular (CV) drugs are well-known VOCC inhibitors used to prevent high blood pressure and to treat hypertension and at the same time might affect chondrogenesis [38]. Recent publications showed that anti-hypertensive VOCC inhibitors can affect the iCa^2+^ level and subsequent expression of the chondrogenic differentiation-related markers in human BMMSCs and animal cartilage-derived chondrocytes [29,34]. However, the effect of an anti-hypertensive cardiovascular therapeutic agent such as nifedipine on iCa^2+^ regulation through the VOCC channels and its effect on chondrogenic differentiation of various types of MSCs has not been investigated so far. The alteration of Ca^2+^ entry into the cytoplasm by prescribed cardiovascular anti-hypertensive Ca^2+^ ion channel blockers might stimulate or suppress the chondrogenic differentiation potential of MSCs in vitro showing a possible regulation mechanisms of OA pathogenesis in vivo. 

Therefore, the aim of this study was to investigate the role of iCa^2+^ and its regulation through the inhibition of VOCC channels with CaV1.2 antagonist nifedipine on chondrogenic differentiation potential of human MenSCs, BMMSCs and OA chondrocytes. The chondrogenic potential of OA cartilage-derived chondrocytes will reveal the possibility to stimulate the regenerating potential of OA cartilage by externally used chemical drugs. 

## 2. Results

### 2.1. The Effects of Nifedipine and BayK8644 on iCa^2+^ Levels in MenSCs, BMMSCs and Chondrocytes

**Figure 1:**
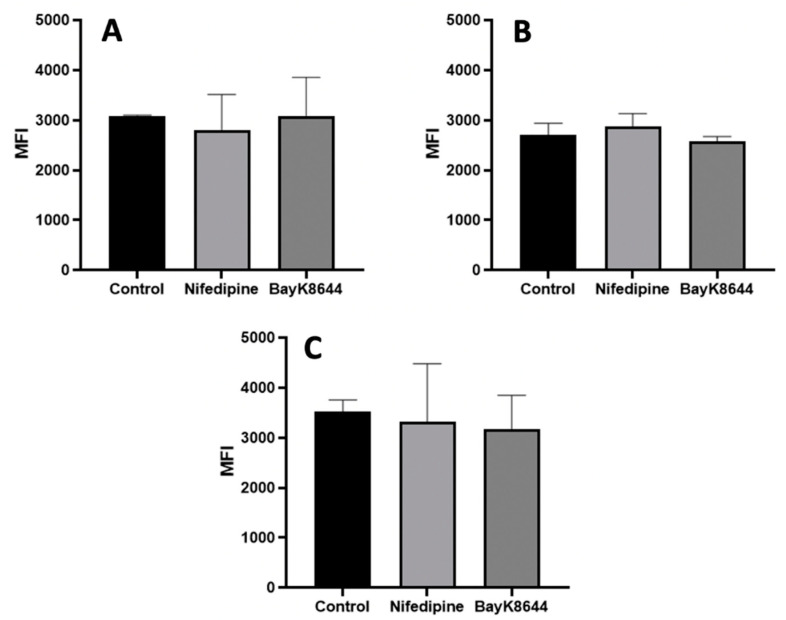
The effects of nifedipine and BayK8644 on iCa^2+^ level in MenSCs (**A**), BMMSCs (**B**) and chondrocytes (**C**) cultivated in a monolayer. Cells were affected with nifedipine (10 μM) or BayK8644 (10 μM) for the 3 days, stained with Cal-520, and mean fluorescence intensity (MFI) was measured by flow cytometer (BD FACSAria). Control–cells without nifedipine or BayK8644. Data are shown as mean ± SD, *n* = 3 different donor samples-derived cells of each type.

**Figure 2:**
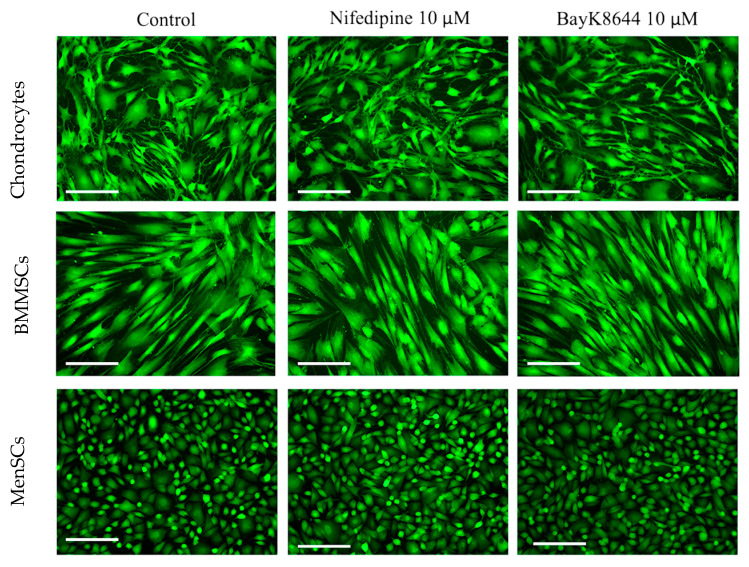
The iCa^2+^ levels in MenSCs, BMMSCs and chondrocytes cultivated in a monolayer, affected with nifedipine (10 μM) or BayK8644 (10 μM) for the 3 days and stained with Cal-520 dye. Control–cells without nifedipine or BayK8644. Cells were visualized with EVOs fluorescent microscope; the representative micrographs are shown. Scale bar 200 μm.

The effects of nifedipine, a VOCC channel CaV1.2 subunit antagonist, was analyzed by flow cytometry (Figure 1) and visualized microscopically (Figure 2). In addition, the effect of the VOCC channel CaV1.2 subunit agonist BayK8644 on iCa^2+^ was also analyzed. The highest iCa^2+^ level was observed in chondrocytes, and the lowest in BMMSCs. The affecting cells with 10 μM of nifedipine or 10 μM of BayK8644 for the 3 days did not significantly change the iCa^2+^ levels (Figure 1).

The identification of iCa^2+^ by fluorescent dye Cal520 and microscope showed almost similar levels of iCa^2+^ in all types of the tested cells (Figure 2). It is probably understandable since microscopical identification is a less sensitive qualitative measurement than flow cytometer. 

However, the iCa^2+^ staining with fluorescent Cal520 dye and time laps measurement revealed a different intensity of iCa^2+^ oscillations in different cells (Figure 2, Supplementary Videos S1–S3). The most intensive iCa^2+^ oscillations were observed in MenSCs as compared to the other cell types (Supplementary Video S1). The frequency of iCa^2+^ oscillations in BMMSCs (Supplementary Video S2) and chondrocytes (Supplementary Video S3) were considerably lower as compared to the MenSCs, suggesting the slower intracellular mechanisms regulating iCa^2+^ release/accumulation in the cytoplasm in those types of cells. In addition, the light microscopy micrographs also showed that BMMSC were the largest in size while MenSCs were the smallest that could also have an impact in iCa^2+^ regulation, oscillations and cell activity/proliferation.

### 2.2. The Release of iCa^2+^ from Intracellular Stores in MenSCs, BMMSCs and Chondrocytes

**Figure 3:**
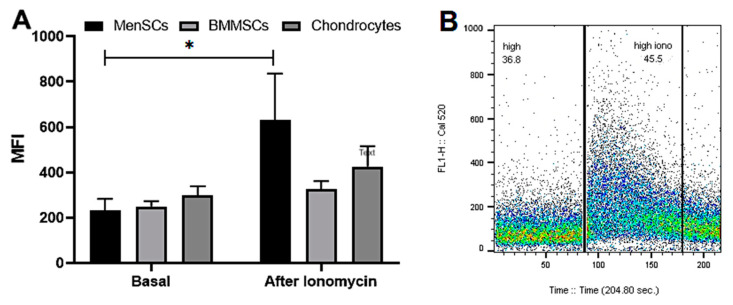
The ionomycin-induced iCa^2+^ release in MenSCs, BMMSCs and chondrocytes. Cells were stained with Cal-520 and analyzed with flow cytometer (Calibur). (**A**) The iCa^2+^ fluorescence is shown as mean fluorescence intensity (MFI) ± standard deviation (SD) before and 90 s after adding the ionomycin (1 μM). * Data are shown as mean ± standard deviation (SD) and are considered significant at *p* ≤ 0.05 (*n* = 5). (**B**) The representative cell population measurement before and after adding the ionomycin (1 μM at 90 s). The two-way ANOVA was used to compare cells.

For the investigation of ionomycin-induced iCa^2+^ release from ER and other intracellular stores, the flow cytometer Calibur was used, which allows for the measurement of the instant fluorescence responses from the fluorescent dye Cal520 labeled cells. The obtained data (Figure 3A) confirmed the results presented in Figure 1 showing the highest basal level of iCa^2+^ in chondrocytes as compared to the other cell types. 

Although the differences in iCa^2+^ basal levels were relatively small between the MenSCs, BMMSCs and chondrocytes, the ionomycin upregulated the iCa^2+^ level significantly stronger in MenSCs compared to the other cell types, suggesting a bigger or more active iCa^2+^ accumulation in those cells’ calcium stores (Figure 3A,B).

### 2.3. The Effect of Nifedipine on Basal Levels and Release of iCa^2+^ from Intracellular Stores of MenSCs, BMMSCs and Chondrocytes

**Figure 4:**
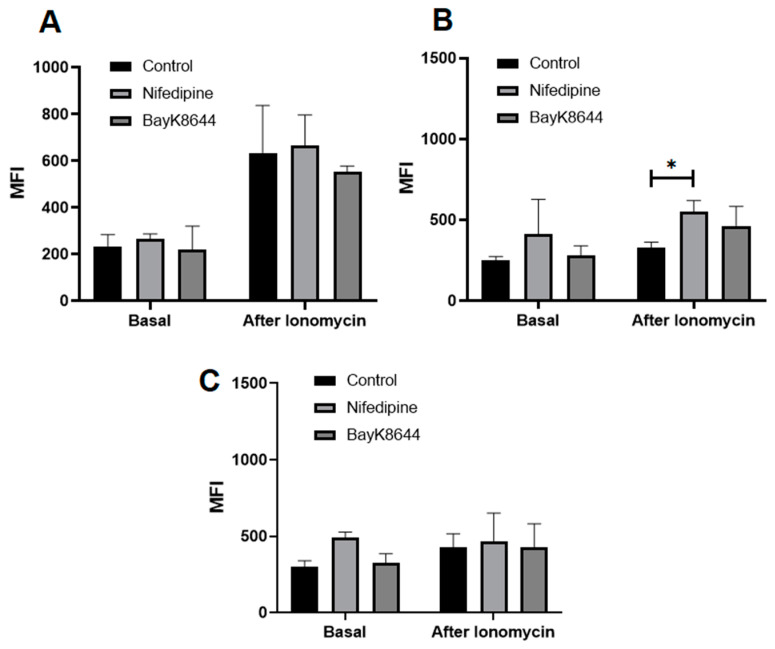
The effect of nifedipine and BayK8644 on the iCa^2+^ levels in MenSCs (**A**), BMMSCs (**B**) and chondrocytes (**C**). Cells were treated with nifedipine (10 μM) or BayK8644 (10 μM) for the 24 h and stained with Cal-520 dye. The accumulation of iCa^2+^ is shown as median fluorescence intensity (MFI) ± standard deviation (SD) before and 90 s after adding ionomycin (1 μM), measured by flow cytometer Calibur. * Data are shown as mean ± standard deviation (SD) and are significant at *p* ≤ 0.05 (*n* = 3) measuring three different donor cells of each type, two-way ANOVA was used.

Since the application of ionomycin resulted in a significantly higher level of iCa^2+^ in MenSCs, as compared to the other types of cells (Figure 3), we further investigated whether the iCa^2+^ store levels in all three cell types can be changed by their preincubation with nifedipine and BayK8644 for the 24 h. Data show the ionomycin-induced release of calcium from the intracellular stores remained to be the highest in MenSCs, as compared to the other types of cells, while it was not considerably altered by the incubation with nifedipine or BayK8644 (Figure 4A). 

Surprisingly, the incubation of BMMSCs with nifedipine for the 24 h resulted in a significantly upregulated level of ionomycin-induced iCa^2+^ release from the intracellular stores (Figure 4B). The iCa^2+^ increase in BMMSCs by adding BayK8644 and ionomycin was also observed. The effect of nifedipine in chondrocytes was similar to the other cells, i.e., nifedipine significantly increased the basal iCa^2+^ level as well as induced slight iCa^2+^ release by ionomycin (Figure 4C). 

These data suggest that MenSCs accumulate a high level of iCa^2+^ in the intracellular stores that were not significantly affected by the VOCC regulators. The accumulation of iCa^2+^ in the intracellular stores of BMMSCs and chondrocytes was considerably lower than in MenSCs. However, the inhibition or stimulation of VOCC with nifedipine or BayK8644, respectively, upregulated the iCa^2+^, particularly in the BMMSCs. Those observations imply that VOCC blocker nifedipine might affect not only CaV1.2 channels present in the plasma membrane but also channels inside the cells. There is also a possibility that nifedipine induces an external Ca^2+^ compensatory inflow through the other channels and/or receptors. 

### 2.4. The Effect of Nifedipine on Proliferation of MenSCs, BMMSCs and Chondrocytes 

**Figure 5:**
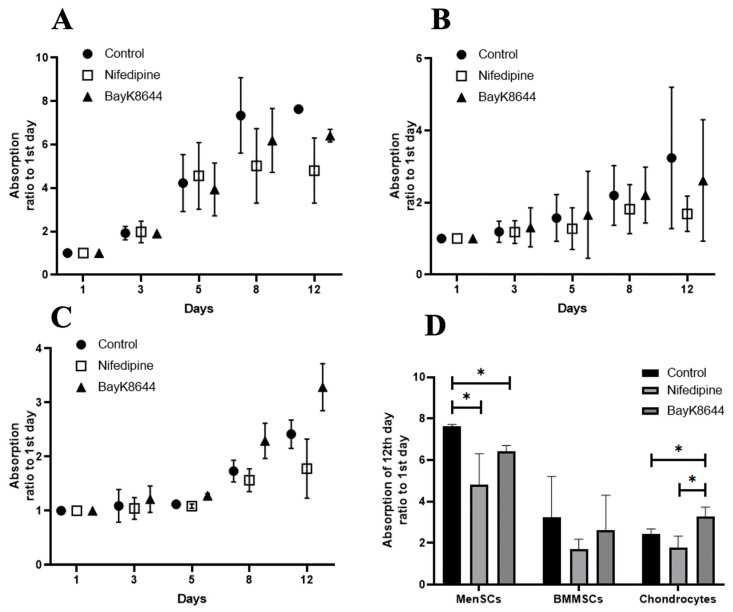
The effect of nifedipine and BayK8644 on MenSCs, BMMSCs and chondrocytes proliferation. Proliferation of MenSCs (**A**), BMMSCs (**B**) and chondrocytes (**C**) after the treatment with nifedipine (10 μM) and BayK8644 (10 μM) for 1, 3, 5, 8, and 12 days. Proliferation was measured using CCK-8 assay. Proliferation rate is shown as a ratio of CCK-8 absorbance (450 nm) to the 1st day of cell adherence. (**D**) The effect of nifedipine on the 12th day of cell proliferation. * Data are shown as mean ± standard deviation (SD) and are significant at *p* ≤ 0.05 (*n* = 3) measuring three different donor samples-derived cells of each type using a two-way ANOVA.

Cell proliferation is an active process that requires a high level of iCa^2+^ [39], energetic and metabolic resources. Since VOCC antagonist nifedipine contributed to the iCa^2+^ accumulation during the 24 h of incubation, we have measured the effect of nifedipine on cell proliferation during 12 days of incubation (Figure 5). Data in Figure 5 show that MenSCs cells more intensively proliferated compared to the other types of cells that could be related to the bigger iCa^2+^ storages.

The VOCC antagonist nifedipine significantly suppressed the proliferation rate of all tested cells (Figure 5A–C). The strongest proliferation inhibiting effect of nifedipine was observed on the most intensively proliferating MenSCs, as compared to the other cells (Figure 5D). Surprisingly, BayK8644 also suppressed the proliferation of MenSCs and BMMSCs, but not in chondrocytes (Figure 5D). Data suggest that iCa^2+^ regulating channels are most active in MenSCs, compared to the other cells. There is also a possibility that certain levels of iCa^2+^ are necessary for the cell proliferation, which is differently regulated in different cells. 

### 2.5. The Effect of Nifedipine on CaV1.2 Level in MenSCs, BMMSCs and Chondrocytes

**Figure 6:**
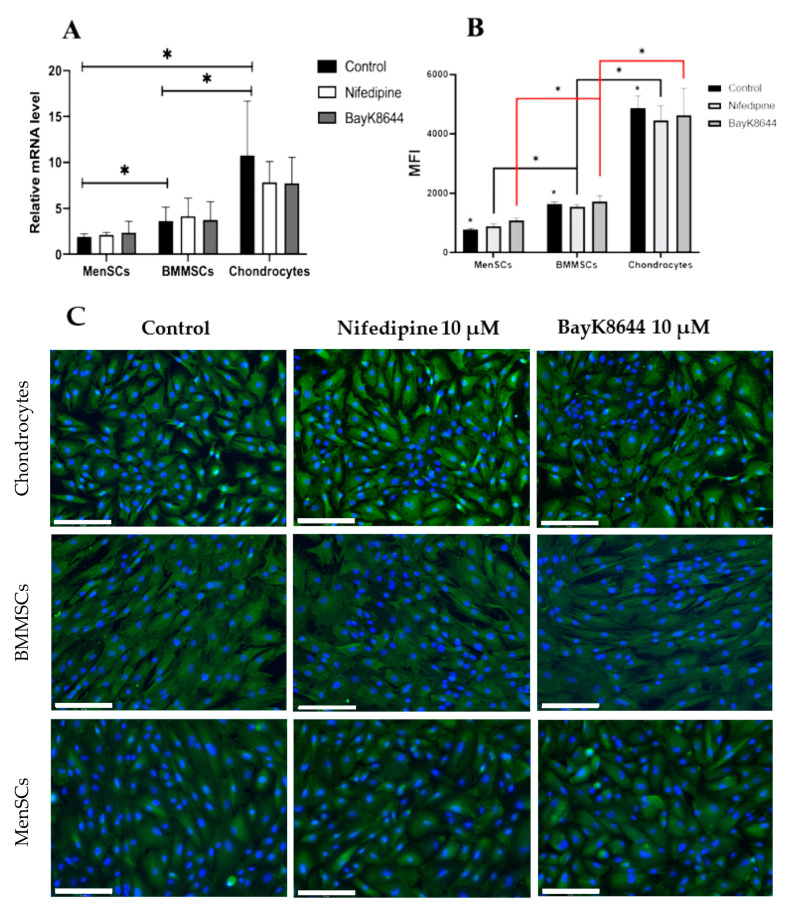
The effects of nifedipine and BayK8644 on CaV1.2 expressions in MenSCs, BMMSCs and chondrocytes. (**A**) The *CACNA1C* gene expression. Relative transcript level was evaluated comparing to the housekeeping *B2M*, *RPS9* and *ACTB* genes and are significant at *p* ≤ 0.05. (**B**) CaV1.2 protein level measured by flow cytometer as mean fluorescence intensity (MFI); data are shown as mean of MFI ± standard deviation (SD). (**C**) Fluorescent micrographs of CaV1.2 in MenSCs, BMMSCs and chondrocytes after the stimulation with nifedipine (10 μM) and BayK8644 (10 μM) for the 3 days. Control–cells without any treatment. Cells were visualized with EVOs fluorescent microscope; the representative micrographs are shown; scale bar = 150 µM. * Data are shown as mean ± standard deviation (SD) and are significant at *p* ≤ 0.05 (*n* = 3) measuring three different donor sample-derived cells of each type using a two-way ANOVA.

Since the VOCC antagonist nifedipine affected the iCa^2+^ accumulation in storages and cell proliferation, the effect of nifedipine on the total level of CaV1.2, a main subunit of the VOCC channel, was also investigated at gene (*CACNA1C*) (Figure 6A) and protein (Figure 6B,C) levels using flow cytometer (BD FACSAria) (Figure 6B) and fluorescent microscope EVOS (Figure 6C). 

Surprisingly, the expression of *CACNA1C* in MenSCs was the lowest, while in chondrocytes was the highest (Figure 6A). The highest *CACNA1C* expression in chondrocytes could be related to their highest level of iCa^2+^ compared to the other cells. No significant change in *CACNA1C* gene expression was observed after the cell’s treatment with nifedipine and BayK8644 for the 3 days. 

The gene expression data were supported by the flow cytometry measurement, which was expressed as mean fluorescence intensity (MFI) (Figure 6B). Similar to the *CACNA1C* gene expression, the level of CaV1.2 was significantly lower in the MenSCs, as compared to the BMMSCs and OA chondrocytes (Figure 6B). Additionally, the cell incubation with nifedipine or BayK8644 for the 3 days did not significantly change the level of CaV1.2 in all measured cells. 

Finally, the level of CaV1.2 in the cells was confirmed by fluorescent micrographs and was highest in chondrocytes, as compared to the BMMSCs and MenSCs (Figure 6C). No significant effect of nifedipine or BayK8644 on CaV1.2 was observed within the 3 days of incubation. 

### 2.6. Different Effects of Nifedipine and BayK8644 on Expression of Chondrogenic Differentiation Markers in MenSCs, BMMSCs and Chondrocytes 

**Figure 7:**
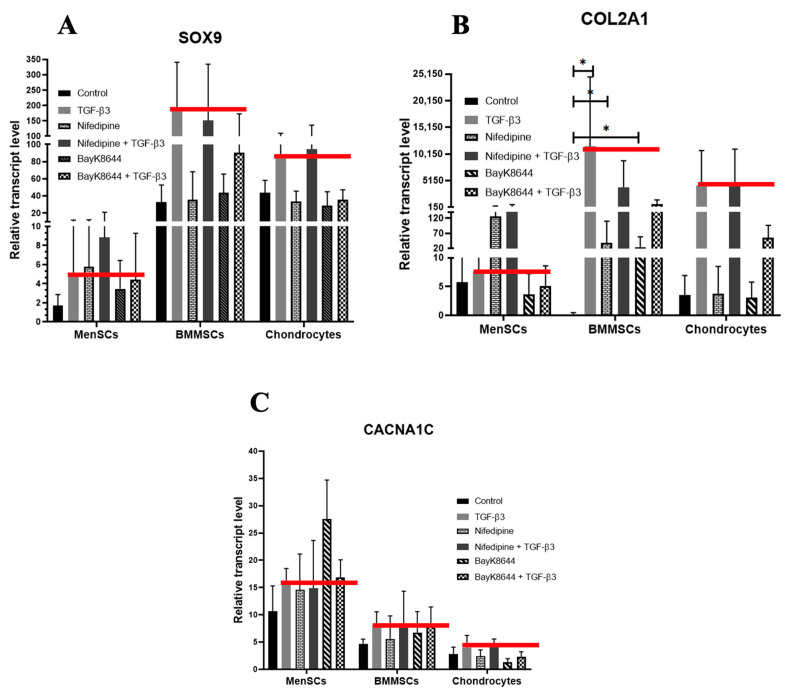
The effect of nifedipine and BayK8644 on chondrogenic differentiation-related genes in MenSCs, BMMSCs and chondrocytes. The expression of *SOX9* (**A**), *COL2A1* (**B**) and *CACNA1C* (**C**) after the stimulation of chondrogenic differentiation for the 21 days with TGF-β3 (10 ng/mL), nifedipine (10 μM) or BayK8644 (10 μM). Control cells were cultured under the same chondrogenic differentiation conditions, but without TGF-β3, nifedipine or BayK8644. Relative transcript level evaluated according to the housekeeping genes *B2M* and *RPS9*. Red lines represent the gene expression after the TGF-β3 stimulation. Data are shown as mean ± standard deviation (SD) and were significant at * *p* ≤ 0.05.

Finally, the effect of nifedipine on chondrogenic differentiation of tested cells was observed. The expression of chondrogenic differentiation identifying genes *SOX9*, *COL2A1* and *CACNA1C* in all types of the cells following chondrogenic differentiation for the 21 days are shown in Figure 7. 

The basal expression of *SOX9*, a transcription factor regulating chondrogenic differentiation, was the lowest in MenSCs, as compared to the other cells (Figure 7A), while the stimulatory effect of TGF-β3 on *SOX9* expression was stronger in BMMSCs and chondrocytes (red line). The VOCC antagonist nifedipine had some stimulating effect on *SOX9* expression in MenSCs with and without TGF-β3, but not in other cell types (Figure 7A). The VOCC agonist BayK8644 suppressed TGF-β3 -induced upregulation of *SOX9* expression in all tested cells.

The basal expression level of *COL2A1*, the most important biomarker of chondrogenic differentiation, was low in all undifferentiated cells. Similarly to the *SOX9* expression, it was stimulated by TGF-β3 in BMMSCs and chondrocytes but not in MenSCs (red line). The nifedipine also stimulated *COL2A1* expression in MenSCs but not in other types of the cells (Figure 7B).

The expression of VOCC subunit CaV1.2 (*CACNA1C)* was also analyzed in all cell types during their chondrogenic differentiation for the 21 days (Figure 7C). The basal expression level of CaV1.2 (*CACNA1C*) at the control level, opposite to the *SOX9* and *CACNA1C* expressions, was the highest in MenSCs and the lowest in OA chondrocytes (Figure 7C). However, neither nifedipine nor BayK8644 had any significant effect on *CACNA1C* expression with or without TGF-β3. The cell stimulation with TGF-β3 also resulted in tendencies of increase in *CACNA1C* gene expression in all cell types. 

Taken together, the tendencies of *SOX9* and *COL2A1* expressions after the TGF-β3 stimulus were similar in all types of cells, i.e., the strongest in BMMSCs and weakest in MenSCs, whereas CaV1.2 antagonist nifedipine had a stimulating effect only on MenSCs. The expression of CaV1.2, opposite to the *SOX9* and *COL2A1* expressions, was highest in MenSCs than in the rest cells. The agonist BayK8644 did not have a chondrogenic differentiation stimulating effect in all cells. Data show that BMMSCs have a highest chondrogenic differentiation potential compared to other tested cells, while the nifedipine stimulated this potential only in MenSCs. The best chondrogenic differentiation potential of BMMSCs might be related to their lower level of iCa^2+^ and/or different regulation of Cav1.2 channel compared to the other cells. Data also suggest that in Ca^2+^ entrance to the cell’s trough, the CaV1.2 is essential for the cell proliferation but not for the chondrogenic differentiation. 

## 3. Discussion

The various types of the multipotent and pluripotent stem cells, tissue engineering technologies, including scaffolds or hydrogels, have been investigated for the cartilage tissue regenerating purposes [40,41]. The multipotent MSCs derived from the adipose [20], human synovial membrane [21], autologous and allogeneic bone marrow [22,23] and chondrocytes [24,25], umbilical cord [26] and other tissues in patients and animal models have been investigated for the OA regeneration purposes. However, it is difficult to compare the efficiency of all cell-based cartilage regeneration model systems investigated both in vitro and in vivo due to the high variation of applied experimental conditions. Beside the direct MSCs participation in cell differentiation processes, they can secret paracrine factors and extracellular vesicles also participating in the chondrogenic differentiation [42]. The paracrine effects of the MSCs even raised a question to change their name to the signalling cells, which did not correspond with all the MSCs functions [14]. 

In this study, we have chosen to investigate the chondrogenic differentiation potential of adult tissue-derived mesenchymal stem/stromal cells (MSCs) such as MenSCs and BMMSCs in comparison to the OA cartilage-derived chondrocytes and to investigate the impact of VOOC channel regulators on it. Previously published studies showed the chondrogenic potential of BMMSCs being high enough to be used for the cartilage regeneration purpose in vitro [43,44]. Our recently published findings also confirmed the higher chondrogenic potential of BMMSCs than other MSCs and their suitability for the investigation of chondrogenic differentiation mechanisms in model systems in vitro [41,45]. Human BMMSCs recruitment to the IL-8- and MIP-3α-containing scaffolds enhanced tissue regeneration of an osteochondral defect site in beagle knee articular cartilage [46]. Moreover, the pre-clinical study of intra-articular injections of BM-MSCs for the treatment of knee osteoarthritis showed positive results [47].

Another type of MSCs used in this study, such as menstrual blood-derived cells, was chosen as an easily accessible source of MSCs. They were firstly isolated and observed by [48]. From that time, the menstrual blood has attracted huge scientific interest, leading to a permanently increasing number of different research studies and possible applications of MenSCs in both experimental and clinical practices. In addition, the MenSCs, due to their origin, could have a higher level of stemness and better differentiation potential [49]. It has been shown that MenSCs, similar to other types of MSCs, possess MSCs-typical qualities such as high proliferative potential, self-renewal property and a multipotent differentiation into osteogenic, adipogenic and chondrogenic directions [48]. Both, MenSCs and BMMSCs can differentiate not only into a mesenchymal origin direction (osteocytes, chondrocytes, adipocytes, muscle cells) but also into the other tissue-specific cells [50,51]. Finally, the articular regeneration potential of OA cartilage-derived chondrocytes has also been investigated, applying the most popular anti-hypertensive pharmacological medications regulating the intracellular calcium level through the VOOC channels.

Calcium is a ubiquitous intracellular signaling molecule controlling various cellular processes such as cell growth and differentiation, secretion of paracrine factors, gene expression, activation of various signaling pathways regulating proper cell and tissue functioning [52]. Calcium signaling has been studied in various cells in order to understand the molecular mechanisms of many physiological and pathophysiological processes such as cancer, diabetes, neuro, muscle and other disorders [53,54,55,56]. The functional effects of iCa^2+^ have been mostly investigated on excitable cells such as neurons and cardiomyocytes [57,58,59,60], while in non-excitable cells such as various types of mesenchymal stem/stromal cells or chondrocytes, the iCa^2+^ signaling modulates cell viability, proliferation and differentiation potentials [61]. Recently, the role of iCa^2+^ and its targeted regulation has been intensively studied in chondrogenesis and OA pathogenesis [32,34]. Since the big part of the antihypertensive drugs are calcium channel blockers, they could also affect chondrocytes and the development of OA, especially in elderly patients, suffering from the hypertension and thus regularly taking antihypertensive medications [38]. 

At the cellular level, the iCa^2+^ can regulate not only the cell proliferation and differentiation but also apoptosis, autophagy, cancer, migration and other intracellular processes [62,63]. It was shown that an extracellular Ca^2+^ concentration is much higher compared to the iCa^2+^, which should be strictly controlled by the network of calcium pumps, transporters, channels and calcium sequestering depo proteins acting differently in different cells [63]. The cell proliferation was shown to be an active cell cycle-related process requiring a certain level of iCa^2+^ and the activation of calcium channels particularly L-type [64]. On the other hand, the iCa^2+^ overload was shown to induce cell apoptosis through the opening of the permeability transition pore and release of apoptogenic factors [65]. The iCa^2+^ level can be also artificially changed by the ionophores such as ionomycin, which helps to release the Ca^2+^ from the intracellular stores to the cell cytoplasm [66,67]. The usage of ionophores to investigate iCa^2+^ changes with or without L-type channel modulators, could be a good model to investigate calcium-related mechanisms regulating MSCs functioning in vitro. 

The results of this study demonstrate that OA chondrocytes cultured under the regular conditions have a slightly higher level of iCa^2+^, as compared to the MenSCs and BMMSCs, while the largest iCa^2+^ store, as well as more intensive iCa^2+^ oscillations were observed in MenSCs. The larger iCa^2+^ stores can also be related to the better MenSCs proliferation and a total differentiation potential [68]. The VOOC antagonist nifedipine or agonist BayK8644 did not affect either the total iCa^2+^ levels nor the intensity of calcium oscillations in all cell types. However, nifedipine affected an intracellular calcium stores in all cells, particularly in BMMSCs that were disrupted by ionomycin. In addition, the nifedipine suppressed all cells proliferation, with the most pronounced effect on MenSCs. The strongest effect of nifedipine on MenSCs proliferation could be explained by the most intensive growth and/or largest iCa^2+^ store better controlling cytoplasmic calcium level as compared to the other cells. Other authors also have shown that nifedipine can initiate the release of iCa^2+^ from ryanodine receptor-mediated endoplasmic reticulum (ER) stores in the neuromuscular junction of neonatal rats [69]. This mechanism might also be explained by the compensatory mechanism of various Ca^2+^ channels in the cells. A similar increase in iCa^2+^ after nifedipine exposure was shown in porcine aortic endothelial cells that do not express L-type VOCCs [70]. Findings suggest that nifedipine might have different mechanisms of action in different cell types and might indirectly stimulate iCa^2+^ levels or release from ER or other stores.

Beside the investigation of nifedipine’s effect on iCa^2+^ levels, stores and proliferation, the regulation of the VOOC sub-channel CaV1.2 and its participation in chondrogenic differentiation is no less important and interesting. The advantage of calcium, as a signaling molecule, over other ions is mainly based on its chemical properties and abundant amount, which requires a strong intracellular control by Ca^2+^-binding proteins, pores and various channels such as L-type, Na/Ca ATPases and others [32,71]. The main L-type channel subunit CaV1.2 plays an important role in activating chondrogenesis in limb development in mouse and chicken embryos and regulating the expression of its downstream genes [72]. It was shown that the inhibition, or even deletion of CaV1.2, downregulated the expression of chondrogenic differentiation-related genes such as *SOX9*, *COL2A1* and aggrecan [72,73]. It has been also demonstrated that the level of CaV1.2 was significantly elevated in the rat dental pulp MSCs during chondrogenic differentiation and its C-terminal domain was essential for this process [74]. 

In our study, the expression of CaV1.2 at protein and gene levels was higher in chondrocytes compared to the other cells and was not changed after the incubation with nifedipine or BayK8644 for 3 days in all three cell types. We assume the cells restore sufficient/excessive iCa^2+^ level by regulating other calcium channels, rather than changing VOCC CaV1.2 expression. The expression of CaV1.2 in OA chondrocytes correlates with their higher level of iCa^2+^ compared to the other cells. 

However, under the chondrogenic differentiation, the expression of the CaV1.2 gene was higher in the MenSCs than in BMMSCs and OA chondrocytes and inversely correlated with the chondrogenic markers such as transcription factor *SOX9* and structural protein *COL2A1* in all cells. Moreover, the different effect of nifedipine on the chondrogenic gene expression in three cell types was observed, i.e., the nifedipine considerably stimulated *SOX9* and *COL2A1* expression in MenSCs with and without TGF-β3, while in BMMSCs and OA chondrocytes it did not. This might be associated with the higher activity, not a level, of CaV1.2 and iCa^2+^ stores in MenSCs than other cells, which was not sufficiently suppressed during the chondrogenesis, thus negatively affecting cell capacity to produce cartilage-related proteins. Data further support the hypothesis that in different cells, the iCa^2+^ is differently regulated, affecting chondrogenic differentiation. Data also suggest that chondrogenic differentiation does not require a high iCa^2+^ level or CaV1.2 activity. The targeted regulation of the iCa^2+^-related mechanism in various cell types could regulate their chondrogenic differentiation potential. 

## 4. Materials and Methods

### 4.1. The Cell Isolation and Culture

MenSCs, BMMSCs and chondrocytes were isolated and cultured as previously described [34,68]. Briefly, menstrual blood samples were collected from the 25–30 years old female donors (*n* = 5) using sterile silicone cups (iCare, China) during the second day of menstruation and MenSCs were isolated using gradient centrifugation. Bone marrow and human articular cartilage samples were collected after surgical procedures in Santaros hospital (Vilnius, Lithuania). BMMSCs were extracted from the 28–32 years old female bone marrow (*n* = 5) using a sterile scalpel, centrifuged and filtered into a sterile tube, while chondrocytes were isolated from the 40–82 years old female cartilage (*n* = 5) using enzymatic digestion by pronase (Roche diagnostics, Basel, Switzerland), followed by type II collagenase (Biochrom AG, Berlin, Germany). All cell types were seeded into cell culture flasks.

All procedures with the donor tissues were performed in accordance with the Bioethical Permission (No. 158200-14-741) and its supplemented version (Permission No. 158200-741-PP2-34) approved by the Vilnius Regional Biomedical Research Ethics Committee.

MenSCs, BMMSCs and chondrocytes were cultivated under the same conditions: in DMEM (1 g/L glucose), supplemented with 10% fetal bovine serum (FBS) (Merck, Rahway, NJ, USA) and 1% penicillin 10,000 units/mL—streptomycin 10,000 μg/mL (PS) (Sigma Aldrich, St. Louis, MO, USA) (named as complete growth medium), 37 °C incubator with 5% CO_2_. For the cell expansion prior to the experiments, 1 ng/mL of fibroblast growth factor-2 (FGF2) (Thermo Fischer Scientific, Waltham, MA, USA) was added to the MenSCs and BMMSCs medium to maintain their stem cells potential and to avoid spontaneous differentiation. Medium for all three types of the cells was changed twice a week. 

MenSCs and BMMSCs were characterized according to typical MSC surface marker expression (CD44, CD73, CD90, CD105, CD14, CD34, CD36, CD45) and ability to differentiate into adipogenic and osteogenic lineages in our previous publication [68].

### 4.2. Measurement of Intracellular Calcium (iCa^2+^) by Fluorescent Microscope and Flow Cytometry

For the iCa^2+^ with the fluorescent microscope, the cells were seeded into 24-well plates at a density of 10,000 cells/well. After the cells reached confluence, they were treated with 1 μL/mL of DMSO (Control) (Sigma Aldrich, St. Louis, MO, USA), 10 μM of nifedipine (Sigma Aldrich, St. Louis, MO, USA) and 10 μM of BayK8644 (Sigma Aldrich, St. Louis, MO, USA) in complete growth medium for 3 days. Nifedipine and BayK8644 were dissolved in DMSO. Then the cells were stained with calcium specific fluorescent dye Cal-520 (1 μM) (Santa Cruz, Biotechnologies, Dallas, TX, USA) in Hanks’ buffered saline solution (HBSS) with probenecid (1 mM) (Sigma Aldrich, St. Louis, MO, USA) and Pluronic^®^ F-127 (0.02%) (Sigma Aldrich, St. Louis, MO, USA) and incubated at +37 °C with 5% CO_2_ for 90 min. After this, the cells were washed twice with PBS and HBSS with 1 mM probenecid (Sigma Aldrich, St. Louis, MO, USA) was added, and cells were analyzed with a fluorescent microscope (EVOS M7000). The intracellular calcium oscillations were recorded, and the record speed was increased by 8-fold (8 frames/s) for the better observation of calcium level changes in individual cells (Supplementary Videos S1–S3). 

For the iCa^2+^ analysis with flow cytometry, the cells were seeded into 6-well plates at a density of 50,000 cells/well. After the cells reached confluence, they were treated with 10 μM of nifedipine (Sigma Aldrich, St. Louis, MO, USA) and 10 μM of BayK8644 (Sigma Aldrich, St. Louis, MO, USA) in complete growth medium for 3 days. Then the cells were detached with trypsin and 50,000 of the cells of each group (incubated with nifedipine and BayK8644, and not incubated (control)) were transferred into flow cytometry tubes, centrifuged at 600× *g* for 5 min and stained with calcium specific fluorescent dye Cal-520 (1 μM) (Santa Cruz, Biotechnologies, Dallas, TX, USA) in DMEM medium with 10% of FBS at +37 °C with 5% CO_2_ for 30 min. After incubation, cells were washed with PBS and centrifuged at 600× *g* for 5 min. The supernatant was discarded, and cells were resuspended in 300 μL of PBS with 1% of BSA. Each sample was run in triplicates; fluorescence was measured using flow cytometer (FACSAria III, Becton Dickinson), 488 nm laser paired with a 530/30 nm bandpass filter. 

In order to measure the store-related iCa^2+^ changes, the cells were treated with nifedipine and BayK8644 for 24 h, divided into flow cytometer tubes, stained with the Cal520 dye, as previously described, and the basic iCa^2+^ level was measured with a Calibur cytometer. This flow cytometer allows for the measurement of instant iCa^2+^ changes. After 90 s of measurement, the 1 μM of ionomycin, iCa^2+^ releasing ionophore, was added (Santa Cruz Biotechnology, Dallas, TX, USA) and the iCa^2+^ change was recorded for ~200 s. The data were analyzed using FlowJo software, version 10 (FlowJo Corp., Ashland, OR, USA). The median fluorescence intensity (MFI) of all samples was calculated by subtracting the MFI of non-stained cells from the MFI of Cal-520 stained cells. Before the addition of ionomycin, measured MFI of the cells was held as basal iCa^2+^ and after 90 s of adding ionomycin—as a high level of iCa^2+^.

### 4.3. Detection of CaV1.2 by Immunocytochemistry

For the CaV1.2 measurements by immunocytochemistry, the cells were seeded on glass cover slips and grown in Petri dishes with complete medium, at a density of 10,000 cells/cover slip. After several days of cultivation, cells were transferred to a new Petri dish, washed 3 times with PBS (Biochrom, Holliston, MA, USA) and fixed with 4% paraformaldehyde (PFA) (Sigma Aldrich, St. Louis, MO, USA) at RT for 20 min. After fixation, the glass slips with the cells were washed twice with PBS and permeabilized with 0.1% Triton X-100 at RT for 20 min. Cells were washed twice with PBS and blocked with 1% bovine serum albumin (BSA) in PBS at +37 °C for 30 min. Antibody against the CaV1.2 subunit (ab84814, Abcam, Cambridge, UK) was prepared in 1% BSA/PBS (1:100) and incubated in 37 °C incubator with 5% CO_2_ for 1 h. Secondary anti-mouse Ig-G1-Alexa488 fluorescent antibody (a21200, Thermo Fischer Scientific, Waltham, MA, USA) was added (1:500) and incubated at 37 °C for 30 min. After that, the cells were washed three times with 1% BSA/PBS solution, stained with 1 µM DAPI (4′,6-diamidino-2-phenylindole) dye for 30–60 s (Vector laboratories, Burlingame, CA, USA), washed with water, put on the objective glass and analyzed with fluorescent microscope (EVOS M7000).

### 4.4. Analysis of CaV1.2 by Flow Cytometer

For the CaV1.2 analysis by flow cytometer, the cells were stained with monoclonal antibody against CaV1.2 (ab84814, Abcam, Cambridge, UK): the cells were enzymatically detached and 50,000 of the cells per one sample were fixed with 4% paraformaldehyde (PFA) for 20 min at RT. After this, cells were washed with PBS and centrifuged at 500× *g* for 5 min. Later on, the cells were permeabilized with 0.1% Triton X-100 at RT for 20 min. and washed once with 1% BSA in PBS, centrifuged and the supernatant was discarded, the cells were blocked with 1% of BSA in PBS solution at RT for 30 min. After this, primary antibody (1:1000) was added and incubated at RT for 1 h. Cells were washed twice with 1% BSA in PBS solution and incubated with fluorescent secondary anti-mouse IgG-PE antibody (P852, Thermo Fisher Scientific, Waltham, MA, USA) and incubated at RT for 30 min. After incubation, the cells were washed twice with and measured in 200 μL of PBS solution containing 1% of BSA. At least 10,000 events were collected by the flow cytometer FACSAriaIII and analyzed using FlowJo analysis software, version 10.

### 4.5. Proliferation Assay

The cells were seeded into 12-well plates at a density of 20,000 cells/well in a complete medium. The next day, cells were divided into 3 treatment groups: control (with dimethyl sulfoxide (DMSO), which is a solvent for nifedipine and BayK8644, and the same amount as nifedipine and BayK8644 was added), nifedipine (10 μM) and BayK8644 (10 μM). Cell proliferation was determined at days 1, 3, 5, 8 and 12 with a cell counting kit—8 (CCK-8) (Dojindo, Munich, 152 Germany) according to the manufacturer’s instructions. The commercial CCK-8 kit allows for the measurement of cell proliferation by utilizing highly water-soluble tetrazolium salt. This salt is being reduced by living cell dehydrogenases and produces soluble orange formazan dye. This way, the amount of the formazan dye generated by dehydrogenases in cells is directly proportional to the number of living cells. The medium was collected in a 96 well plate and absorbance at 450 nm was quantified with a SpectraMax i3 spectrophotometer (Molecular Devices, San Jose, CA, USA).

### 4.6. Chondrogenic Differentiation

The cells were detached, counted and transferred into conical 15 mL tubes (250,000 cells/tube). After that, the cells were washed with DMEM medium without FBS, centrifuged at 600× *g* for 5 min and supernatant was changed to the chondrogenic medium (high glucose (4.5 g/L) DMEM medium) and incubated for 24 h. During this period, the cells formed 3D cell pellets. The next day, chondrogenic medium (high glucose (4.5 g/L) DMEM medium, 1% PS (Merck, Rahway, NJ, USA), 1% insulin-transferrin-selenium (ITS) (Gibco Life Technologies, Grand Island, NY, USA), 0.35 mM L-proline (Carl Roth, Karlsruhe, Germany), 10^−7^ M dexamethasone, 0.17 mM ascorbic acid phosphate (Sigma Aldrich, St. Louis, MO, USA) with or without 10 ng/mL of TGF-β3 (Thermo Fisher Scientific, Waltham, MA, USA) was added. The chondrogenic differentiation of each sample was evaluated comparing samples with and without growth factor TGF-β3. The chondrogenic medium was changed three times a week.

The effect of nifedipine and BayK8644 on chondrogenesis of MenSCs, BMMSCs and chondrocytes was investigated as follows: 1 μL/mL of DMSO (Control), 10 μM of nifedipine and 10 μM of BayK8644 were added to the cells in complete chondrogenic media and incubated for 21 days, changing chondrogenic medium as usual. After chondrogenic differentiation, the cell pellets were analyzed by RT-qPCR.

### 4.7. Gene Expression Analysis

Cell pellets were washed twice with PBS and homogenized with a syringe in RLT lysis buffer with 10% mercaptoethanol (from Qiagen RNeasy Mini Kit kit, Venlo, Netherlands) according to the manufacturer’s recommendations. The RNA was subsequently purified with RNeasy Mini Spin columns (Qiagen kit, Venlo, Netherlands). RNA concentration and purity was measured with the spectrophotometer (Spectramax i3). 

RNA samples were treated with dsDNase and cDNA synthesis was performed with the Maxima^®^First Strand cDNA Synthesis Kit (Thermofisher Scientific, Waltham, MA, USA) according to the manufacturer protocol. RT-qPCR was performed using Maxima Probe qPCR Master Mix (2X) with the Agilent Aria MX instrument. The TaqMan gene expression assays were used for gene expression analysis. The RT-qPCR reaction volume contained 25 μL of reaction buffer with 1 μL of 20X TaqMan gene expression assay mix. All reactions were run in triplicates. Cycle conditions were as follows: initial denaturation step at 95 °C for 10 min, followed by 40 cycles of denaturation at 95 °C for 15 s and finally annealing and extension at 60 °C for 60 s. Each RNA sample was controlled for genomic DNA contamination by the reactions without reverse transcriptase, and the reagent contamination was checked by the reactions without template (NTC). For the normalization of gene expression, the geometric mean of two reference genes—*RPS9* and *B2M* was used. 

RT-qPCR reaction mixes were prepared with Maxima Probe qPCR Master Mix (Thermo Fischer Scientific, Waltham, MA, USA) and TaqMan Gene expression Assays (*RPS9*—Hs02339424_g1, *B2M*—Hs00984230_m1, *ACTB*—Hs01060665_g1, *SOX9*—Hs00165814_m1, *COL2A1*—Hs01060345_m1, *CACNA1C*—Hs00167681_m1 (Thermo Fischer Scientific, Waltham, MA, USA).

### 4.8. Statistical Analysis

The statistical difference between groups was evaluated using two-way analysis of variance (ANOVA), and Student’s *t*-test was used to calculate statistical significance. Data were considered to be statistically significant at *p* ≤ 0.05. GraphPad Prism 8.4.0 software was used to perform statistical analysis. Not less than three patients’ cells and three repeats were measured.

## 5. Conclusions

Taken together, the adult human tissues-derived MSCs of different origins may not be equally applied for the cartilage regeneration purposes, as they possess different chondrogenic differentiation potential. Data show the BM-MSCs having a higher chondrogenic differentiation potential than MenSCs or OA chondrocytes. Molecular pathways regulating the iCa^2+^ signaling through the VOCC subunit CaV1.2 can affect the proliferation and differentiation of MSCs and OA chondrocytes, including chondrogenic response. Therefore, the stem cell therapies and antihypertensive drugs, such as CaV1.2 inhibitor nifedipine, should be prescribed with cautiousness to the patients with hypertension and/or joint diseases.

## Data Availability

The data supporting these findings can be found at the State Research Institute Centre for Innovative Medicine, Department of Regenerative Medicine.

## Supplementary Materials

The supporting information can be downloaded at: https://www.mdpi.com/article/10.3390/ijms24076730/s1.

## Abbreviations

BMMSCs	bone marrow mesenchymal stem cells
BSA	bovine serum albumin
Ca^2+^	calcium ions
COL2A1	collagen type II gene
DAPI	4′,6-diamidino-2-phenylindole
ER	endoplasmic reticulum
FBS	fetal bovine serum
FGF2	fibroblast growth factor-2
iCa^2+^	intracellular Ca^2+^;
MenSCs	menstrual blood-derived mesenchymal stem cells
MFI	median fluorescence intensity
MSCs	mesenchymal stem cells
NSAIDs	non-steroidal anti-inflammatory drugs
OA	osteoarthritis
PFA	paraformaldehyde
TGF-β3	transforming growth factor β3
VOCC	voltage-operated calcium channels
